# A Hybrid Indoor Localization and Navigation System with Map Matching for Pedestrians Using Smartphones

**DOI:** 10.3390/s151229827

**Published:** 2015-12-05

**Authors:** Qinglin Tian, Zoran Salcic, Kevin I-Kai Wang, Yun Pan

**Affiliations:** 1 College of Electrical Engineering, Zhejiang University, Hangzhou 310027, China;; 2 Department of Electrical and Computer Engineering, University of Auckland, Auckland 1010, New Zealand; z.salcic@auckland.ac.nz (Z.S.); kevin.wang@auckland.ac.nz (K.I.-K.W.); 3 Department of Information Science & Electronic Engineering, Zhejiang University, Hangzhou 310027, China; panyun@vlsi.zju.edu.cn

**Keywords:** pedestrian dead reckoning, hybrid, map matching, drift-free, particle filter

## Abstract

Pedestrian dead reckoning is a common technique applied in indoor inertial navigation systems that is able to provide accurate tracking performance within short distances. Sensor drift is the main bottleneck in extending the system to long-distance and long-term tracking. In this paper, a hybrid system integrating traditional pedestrian dead reckoning based on the use of inertial measurement units, short-range radio frequency systems and particle filter map matching is proposed. The system is a drift-free pedestrian navigation system where position error and sensor drift is regularly corrected and is able to provide long-term accurate and reliable tracking. Moreover, the whole system is implemented on a commercial off-the-shelf smartphone and achieves real-time positioning and tracking performance with satisfactory accuracy.

## 1. Introduction

Indoor Positioning Systems (IPS) for pedestrians, aiming at providing accurate indoor position information for tracking and navigation, are of significant interest for increasing number of Location Based Services (LBS). A number of IPS solutions can be found in literature. Majority of these systems are based on the use of Radio Frequency (RF) and/or Inertial Navigation Systems (INS) sensing techniques.

For RF-based systems, Wi-Fi [1,2,3] signals are the most widely used type due to their extensive coverage. With known anchor points, trilateration, triangulation or fingerprinting is commonly used to obtain location information based on the signal characteristics such as received signal strength indicator (RSSI) [2], angle-of-arrival (AoA) and time-of-arrival (ToA) [4]. These approaches suffer from indoor environment fluctuation and multi-path problems, while signal fingerprinting [3] requires a radio map to be established before deployment. Other short-range RF technologies, including Bluetooth [5], radio-frequency identification (RFID) [6,7,8] and near field communication (NFC) [9,10], are also considered. Methods similar to those used in Wi-Fi are employed for Bluetooth and RFID. The corresponding systems are limited to a small area due to the short-range characteristic of the signal protocol while the effective area can be increased by relying on a dense installation of signal transmitters. The emerging technology of NFC can be used to build an interactive indoor navigation systems [9] where users equipped with NFC enabled devices can navigate by scanning a series of NFC nodes along the route.

Meanwhile, INS [11,12,13,14,15,16,17,18,19,20,21] is a powerful alternative for tracking and navigation, particularly if combined with human kinematics modeling. Pedestrian dead reckoning (PDR) is a common approach used in such systems with an inertial measurement unit (IMU) collecting motion data while the pedestrian is walking. IMUs often integrate several sensors including accelerometers, gyroscopes and magnetometers, and the sensor data are used to determine the distance and orientation the pedestrian has travelled by double integration of acceleration- or step-based approaches. INS is able to deliver accurate tracking performance within a relatively short distance and the errors accumulate as the sensors drift over time. Different approaches are proposed to deal with sensor drift. For example, self-resetting [11] or zero-velocity update (ZUPT) [12] is used to calibrate the drift errors when double integrating the acceleration along the direction of motion over time to calculate the travelled distance. Information fusion from gyroscopes and magnetometers through an extended Kalman filter (EKF) [16] is proposed to obtain correct heading from drifted sensor data. Additional consideration such as map and physical constraints [18] can also help to improve the accuracy of the system. Regarding the INS platform, it can either be dedicated IMU nodes [11,12,13,14,15] or handheld devices [16,17,18,19,20,21] such as smartphones with a built-in IMU. Thanks to the rapid development of mobile devices, smartphones are becoming an ideal and excellent platform for IPS, enabling self-contained systems because of its accessibility and integration of various sensors and powerful processing capability. In smartphone-based INS, step-based approaches [16,17,18,19,20,21] are more commonly used as the drift in the accelerometer has little influence on the whole system and efforts are devoted to accurate step detection, step length estimation and orientation determination.

Both RF-based systems and INS have their strengths and weaknesses. Hybrid systems which incorporate the INS and RF [22,23,24,25,26,27] approaches or proximity-based user interactions [28] were proposed to further enhance the reliability and performance of IPS. Studies [29,30] have also presented the feasibility of fusing GPS with INS to offer long-term accurate positioning performance, but such satisfactory performance is only available outdoors due to the limitations of GPS signals. In this paper, we propose a hybrid indoor localization and navigation (HILN) system for pedestrians combining the advantages of both the RF and INS systems. A step-based PDR system built with IMU only, known as enhanced PDR tracking system and typically providing sub-meter positioning and tracking accuracy, was proposed in our previous work [20]. In this paper, the step-based PDR system using smartphones is extended by fusing a Short range proximity access control system, referred to as SRP, which can be RFID- or NFC-based, and integrated with the constraints in the form of an indoor map. Though SRP is limited by the short range at which a smartphone can communicate with an SRP device, the feature is turned to a strength as the exact position can be obtained when the target to be tracked is within the effective range of an SRP device. By combining with traditional PDR, the exact position obtained can be used to calibrate the error caused by sensor drift in PDR, enabling PDR to offer long-term accurate positioning performance as long as SRP devices are available with reasonable density. At the same time, the PDR system contributes to the reduction of the required density of SRP devices as it compensates the tracking between adjacent SRP nodes. Moreover, contrary to an external SRP network that requires the user to scan nodes/tags interactively, the interaction is naturally integrated with the access control system, which is a common scenario in buildings, especially office and public buildings. Therefore, the proposed HILN system can be readily deployed in buildings equipped with already existing SRP access control systems. The proposed system is capable of providing reliable long-term localization and tracking performance with satisfactory accuracy while operating on off-the-shelf devices. In this case, real-time navigation is achieved on the tracked smartphone itself.

The remainder of the paper is organized as follows: a literature review on related works and the contributions of this paper is presented in Section 2. Section 3 describes the proposed hybrid system in detail. Evaluations and discussions can be found in Section 4 and conclusions and future work are presented in Section 5.

## 2. Related Works

Pedestrian dead reckoning (PDR) is a common approach used in tracking pedestrians. Basically, it consists of two major parts, which are the distance calculation and orientation estimation. The distance travelled can be accumulated through double integration of acceleration and a step-based approach. Accurate estimations of travelled distance are reported in both approaches. When performing double integration, ZUPT [12] is widely applied to reduce the accumulated error caused by drift and noise in accelerometers by identifying the stance phase of walk, while in step-based methods, different step length models [17,19,20,31,32] are developed to enhance the accuracy. In the static step length model [31], the step length is considered as a constant for each individual according to height and gender. Dynamic models are also proposed to evaluate step length for every single step using variables such as vertical acceleration [17,32], leg length combined with height change during walking [19,32] and step frequency [20].

For orientation determination, magnetometers and gyroscopes can be utilized to obtain the heading of the pedestrian. Different methods are proposed to calibrate the heading errors caused by sensor drift or magnetic interference. Zengshan *et al.* [16] proposed an EKF to determine the correct heading by fusing data from both the magnetometer and gyroscope, and [21] also used information fusion from these two sensors to obtain a reliable heading estimation under magnetic disturbance.

Apart from sensor error calibration, physical constraints are also taken into consideration to improve the positioning and tracking accuracy of the system. Particle filter (PF)-based map matching methods are used in [15,17] to eliminate the possibilities of positions outside the range of a valid map and [13] introduced a shape filter to regulate the shape of the path by turn detection, assuming a perpendicular route inside buildings. For [15], improvement incorporating map matching and PF are proposed to enhance the localization accuracy as well as correcting sensors errors with the help of the map. Nevertheless, only the corridor scenario in an indoor environment is considered and the computationally intensive principle component analysis (PCA) algorithm is used to obtain the step orientation.

Meanwhile, an IPS solely based on SRP techniques remains an alternative solution. RFID signals are only able to cover a limited area (within a few meters) while NFC has a working range of up to only 10 cm. Lim et al. [6] proposed a robust RFID-based IPS by deploying 176 tags in the ceiling of the room within an area of 4.2 m × 8.4 m and achieved 97% positioning accuracy within 1 m. Despite its accuracy, the requirement for a dense tag distribution presents a challenge in practice. Ozdenizci et al. [9] developed an interactive navigation system where NFC tags are placed at known positions in a building and the users carrying NFC-enabled devices can navigate by scanning tags one after another on the route to destination with the help of the map.

In addition, hybrid systems fusing INS and RF approaches are addressed in [22,23,24,25,26,27]. Jin and Lee et al. [22,23] implemented a pedestrian localization system by integrating a PDR approach and RF beacon nodes (BNs). Ten BNs installed at known locations in an area of 13.2 m × 20 m are used in [22] to enhance the position accuracy of a PDR system by BN ranging using PF. Five unknown position BNs deployed in a 380 m^2^ environment are used in [23]. The locations of BNs are first determined using PF, then BN ranging is also used to achieve improved localization accuracy. In [24], Wi-Fi RSSI is introduced to enhance the long-term accuracy of PDR by means of recursive density estimation. EKF is used in [25] to fuse the PDR system with Zigbee RSSI to reduce the cumulative errors of PDR. Nevertheless, the BNs and Wi-Fi/Zigbee anchor points are external infrastructure and may incur additional cost. Also, BNs and Wi-Fi/Zigbee signals are vulnerable to environmental fluctuations and these modules significantly increase the power consumption of the overall system. The fusion of INS with SRP approaches are investigated in [26,27]. In [26], NFC readers are attached to the underside of the shoes to detect NFC tags distributed on the floor and thus correct the INS position error. This method suffers from limitations since users must know the exact positions of the tags since the working range of NFC signals is very short. The fusion of INS and RFID ranging is presented in [27] to reduce the error caused by sensor drift and achieve long-term tracking of pedestrians. In order to achieve an average position error of 1.35 m regardless of travelled distance, 71 RFID tags are required in an area of 40 m × 60 m, which represents a significant infrastructure cost incurred by the system. Other than hybrid systems incorporating PDR and RF systems, a PDR system fused with user interaction by scanning Quick Response (QR) code is proposed in [28], which is also proximity based. The position of the pedestrian is corrected to the location indicated by the QR code upon scanning it using a smartphone. The system achieves an average localization error of 0.64 m in an experimental path of 35 m with QR codes placed every 10 m along the route as an additional infrastructure. Further, the drift of kinematic sensors over time is not corrected in [22,23,24,25,26,27,28].

The experiments with the aforementioned techniques are limited to relatively short distances within several hundred meters [13,15,16,17,18,19,20,21,22,23,24,25,26,28], which is a limitation of kinematic sensor-based approaches due to sensor drift over time and the cost for external dedicated infrastructure support.

**Table 1:** Summary of related works with PDR approaches.

	Sensors^*^	Technique^*^	Evaluation Scenario^*^	Max. Distance	Achieved Accuracy^**^
[13]	Acc, Gyro	ZUPT, Map Matching	Waist mounted sensor node	40 m	TTD, 98.26%
[14]	Acc, Gyro	Ramp detection	Foot mounted sensor node	1000 m	ε/TTD, 0.15%–1.06%
[15]	Acc, Mag	PDR with Map Matching	In-pocket motion sensor	104 m	Average LE, 0.55 m–0.93 m
[16]	Acc, Gyro, Mag	Neural network, EKF	Smartphone held in hand in front of body, outdoors	400 m	SD, approx. 100%; TTD, 97.98%–102.67% ε/TTD, 0.85%–2%
[17]	Acc, Gyro	Quaternion complementary filter	Mobile device kept in jacket and trousers pocket, held in hand in front of body	270 m	SD, above 98%; Median of TTD, 100.22%
[18]	Acc, Gyro, Mag	Map Matching, Mag and Gyro Fusion	Smartphone kept in pocket, held in hand while calling, swinging, in front of body	600 m	Average LE, 0.45 m–0.74 m; 95^th^ percentile of LE, 0.8 m–1.71 m
[19]	Acc, Gyro	Novel stride length estimator	Smartphone mounted on waist and kept in chest pocket	6.69 m	TTD, 96.14%–97.35%
[20]	Acc, Gyro	Mode classification	Smartphone kept in trouser pocket, held in hand while swinging and in front of body	96.33 m	SD, 95.49%; TTD, 99.7%
[21]	Acc, Gyro, Mag	Mag and Gyro Fusion	Smartphone held in hand in front of body	168.55 m	Average LE, 1.35 m; Average HE, 2.28^o^
[22]	Acc, Gyro, BN	PDR with BN Ranging	Smartphone held in hand with BNs installed on ceiling	90 m	Average LE, 0.88 m
[23]	Acc, Mag, BN	Estimating BN positions, PDR with BN Ranging	Smartphone held in hand with BNs deployed at arbitrary positions on floor	480 m	Average LE, 1.59 m–5.46 m
[24]	Acc, Gyro, Wi-Fi	PDR with Wi-Fi RSSI fusion by Recursive Density Estimation	Smartphone held in hand with five Wi-Fi access points installed	120 m	Average LE, less than 5.22 m
[25]	Acc, Gyro, Wi-Fi	PDR with Zigbee RSSI fusion by EKF	Waist mounted IMU and Zigbee node	25 m	Maximum LE, 4 m
[26]	Acc, Gyro, NFC	PDR with NFC error correction	Smartphone held in hand in front of body with NFC tags on floor ground	44 m	Maximum LE, 1.7 m
[27]	Acc, Gyro, Mag, RFID	PDR with RFID RSSI fusion by EKF	Foot mounted IMU with RFID tags installed in rooms	1000 m	Average ε/TTD, 1.27%
[28]	Acc, Gyro	PDR with assistive QR code	Smartphone held in hand and scan QR code along the path	35 m	LE, 0.64 m

^*^ Acc = Accelerometer, Gyro = Gyroscope, Mag = Magnetometer, BN = Beacon Node, QR = Quick Response; ^**^ TTD = total travelled distance, ε/TTD = final position error over total travelled distance, SD = step detection, LE = Localization Error, HE = heading error.

Antonio *et al.* [14] proposed a PDR system with ramp detection capable of providing drift-free localization in long-distance walks over one kilometer, but the approach relies on regular ramps located along the path, which significantly limits the practical deployment of the system as ramps are structural features of a building. Also, most experimental results are presented in the corridors of indoor environment only [13,14,18,19,20,21,23,24].

Table 1 summarizes the related works with PDR-based approaches [13,14,15,16,17,18,19,20,21,22,23,24,25,26,27,28] in terms of sensors used, techniques employed, the scenarios in which the systems are evaluated, maximum travelled distance in experiment evaluation and achieved accuracy, respectively. Different criteria to quantify the accuracy of positioning and navigation systems including the estimated total travelled distance (TTD) with respect to ground truth distance, step detection (SD) rate, average and 95th percentile of localization error (LE) and average of heading error (HE) are found in the literature. In addition, when the experimental route has the same starting and ending point, the final position error over total travelled distance (ε/TTD) can be obtained and it equals to the final position drift with respect to the staring/ending point.

To the best of our knowledge, existing hybrid systems combing PDR and RF/SRP approaches rely on dedicated external infrastructure at additional installation and deployment cost [22,23,24,25,26,27] and have limitations such as being vulnerable to environmental fluctuations [24,25]. The sensor drift in inertial sensors, which is the source of error in INS, is not corrected over time as well [22,23,24,25,26,27,28]. Systems based solely on RFID are confined within a limited area and interactive positioning systems using NFC are unable to provide location information between two adjacent nodes, resulting in a need for dense installation of nodes. By combining the advantages of different systems, a hybrid indoor localization and navigation (HILN) system is proposed in this paper, which contributions can be listed as follows:

1. A light-weight PDR approach [20] is fused with a SRP system through user interaction and the accumulated errors, both positioning error and sensor drift, are regularly corrected. Moreover, the SRP system takes advantage of the essence of people’s walking behavior in buildings, *i.e.*, moving from one room to another via corridors, with SRP interaction naturally integrated with existing access control systems at the entrances/exits of rooms, which are commonly found in buildings. It extends the practical scenario of IPS, covering both rooms and corridors of indoor environment. Therefore, the proposed system is able to deliver accurate long-term, distance-independent tracking performance without extra infrastructure installation.

2. The positioning accuracy is further enhanced by a novel simplified PF map matching algorithm with a lost track recovery mechanism. The computational complexity of the overall system is therefore minimized to reduce power consumption and increase real-time capability since the resources of a mobile device is quite limited.

3. The whole system is implemented and validated on an off-the-shelf smartphone, achieving accurate real-time positioning and tracking of pedestrians with long-term robustness and reliability.

The HILN system is a drift-free, low-cost, light-weight, easy-to-integrate IPS, enabling ubiquitous navigation of pedestrians in buildings equipped with access control systems at no additional cost. The accurate position information can then be the basis of all LBS available.

## 3. Proposed HILN System

In this section, the proposed HILN system is introduced first, followed by descriptions of individual components, including the step-based PDR system, adaptive drift calibration by SRP interaction and PF-based map matching algorithm, respectively.

### 3.1. System Overview

**Figure 1:**
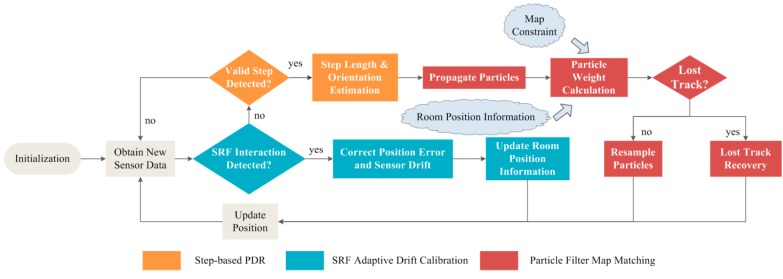
Overview of HILN system.

**Figure 2:**
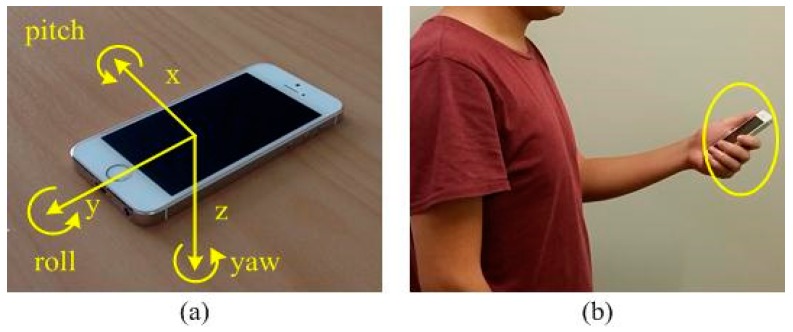
(**a**) Phone axis definition (**b**) Phone position.

Figure 1 presents an overview of the HILN system operation. The axis definition of the smartphone used and the way it is carried during walking are illustrated in Figure 2a,b, respectively. The whole HILN system is implemented by a customized software application on a standard iPhone 5s where sensor data are sampled at 50 Hz. The sensor data used in HILN include 3-axis user acceleration and gravity acceleration in g (9.8 m/s^2^) from the accelerometer and yaw data from the gyroscope in radians. The timestamp of each sample is also recorded. The accelerometer data are used for detecting steps while the step orientation is estimated using the yaw data from the gyroscope. Yaw data provide an indication of the phone’s orientation change around its *z*-axis with respect to a reference frame where the phone’s *x*-*y* plane is horizontal. Since the phone is hand-held in front of body as in Figure 2b, which is a common scenario in indoor environments [16,18,20,21,22,23,26], the yaw samples are a direct indication of step orientation.

The processing iterations aim at updating the pedestrian position upon obtaining new sensor data. Firstly, the SRP-based subsystem checks whether an SRP interaction is detected. Every time an interaction is detected, the position error and sensor drift is corrected by updating the position of the pedestrian to the exact location indicated by the SRP interaction. The room position information (RPI), defining whether the pedestrian is in corridor or a specific room, can also be derived since the interaction is integrated with the access control system when entering or exiting a room. The updated position information provided by the SRP subsystem is assigned with higher priority than the PDR subsystem since the accuracy of the SRP subsystem is guaranteed. If no SRP interaction is detected, the PDR subsystem then performs step detection based on acceleration signals. When no valid step is detected, the processing in current iteration is completed, waiting for new available sensor data. Otherwise, step length and orientation are estimated if a valid step is identified.

The PF map matching subsystem is introduced to enhance the localization and tracking performance of the PDR subsystem. The step length and orientation estimated by the PDR subsystem is used in particle propagation according to human kinematic model. Then, the weights of particles are evaluated combining the map constraints and RPI of the pedestrian. Map constraints are utilized to keep the tracking target in the valid range of the map and the RPI provided by the SRP subsystem is used to further constrain feasible positions. For example, a corridor RPI restricts the position of the pedestrian in the corridor zone only. In order to strengthen the robustness of the map matching subsystem, a lost track recovery mechanism is added, addressing situations where the PF loses track. The position information generated by the PF, which is always in valid map zone, is used to update the location of the pedestrian. The following subsections introduce the individual subsystems in details.

### 3.2. Step-Based PDR System

In a step-based PDR system, the position of a pedestrian is usually presented using a two-dimensional vector [*x*, *y*]*^T^* in a defined coordinate system and is updated from a known initial position according to Equation (1) where [*x_s+1_*, *y_s+1_*]*^T^*, [*x_s_*, *y_s_*]*^T^*, *L_s_* and *θ* denote the updated position, original position before a step, step length and the angle of the step in the coordinate system:

(1) $$\left[\begin{array}{c} x_{\text{s}+1}\\ y_{\text{s}+1} \end{array}\right]=\left[\begin{array}{c} x_{s}\\ y_{s} \end{array}\right]+{\text{L}}_{s}*\left[\begin{array}{c} \text{sin}\theta \\ \text{cos}\theta \end{array}\right]$$

The PDR subsystem includes step detection, step length and orientation estimation.

#### 3.2.1. Step Detection

**Figure 3:**
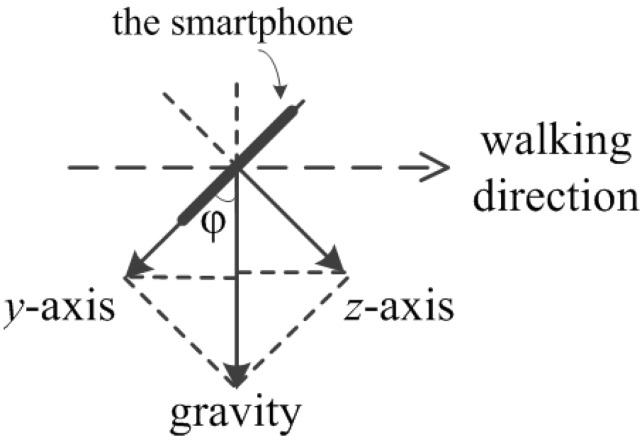
Calculation of vertical acceleration.

Vertical acceleration (perpendicular to the horizontal plane) is used for step detection when the smartphone is carried as in Figure 2b, while periodic vertical displacement of the smartphone coupled to human walking motion is observed. The vertical acceleration is calculated according to Equation (2), Equation (3) where *grav_y_*, *grav_z_*, *uacc_y_*, *uacc_z_* represent the gravitational acceleration along *y* and *z* axis, and user acceleration along *y* and *z* axis, respectively. As can be seen from Figure 3, the cross-section of the plane perpendicular to the ground and parallel to the walking direction, the angle ϕ is firstly computed using Equation (2) and vertical acceleration is then calculated by combining the *y* and *z* axis user acceleration component as in Equation (3). It is observed that different people tend to hold the phone differently with varying angle of ϕ. When calculating vertical acceleration according to Equations (2) and (3), the acceleration can be obtained for an arbitrary value of ϕ within the range of [0, *π*/2], achieving robust performance for different holding preferences of the smartphone:

(2) *ϕ* = *atan*(*abs*(*grav_z_*/*grav_y_*))

*acc_vertical_* = uacc_z_*sinϕ+ uacc_y_*cosϕ (3)

Steps are basically identified by thresholding, *i.e.*, a valid step corresponds to a positive peak whose magnitude is greater than a given threshold. The following constraints to achieve better detection were proposed in our previous work [20]:

1. As human step frequency is in the range of 0.5–5 Hz [17], a low-pass filter with cut-off frequency at 5Hz is applied to the vertical acceleration to filter out noise.

2. Since the maximum step frequency is 5 Hz, the minimum time interval between two consecutive steps is 0.2 s. Every time a valid peak above the threshold is detected, its timestamp is compared to the timestamp of the previous step timestamp, making sure that the minimum time interval constraint between steps is satisfied.

3. False spikes are observed in the processed and filtered acceleration signal that appears before the true signal peak of a valid step. A false peak rejection (FPR) mechanism [20] is added to filter out the false peaks. A positive peak exceeding the threshold is considered as a valid step when no negative peak is found in the following *n_fpr_* acceleration samples, where *n_fpr_* denotes the size of FPR window in a number of samples. The value of *n_fpr_* is currently set to six based on our previous observations reported in [20].

#### 3.2.2. Step Length & Orientation Estimation

Step length is calculated according to the model in [20], which is proportional to height and step frequency (steps per second) as shown in Equation (4). *k* is a constant value taking gender into consideration and equals 0.3139 for males or 0.2975 for females. The same values for *k* as in [20] are used to achieve optimal accuracy of step length estimation where performances of different step length models are experimentally evaluated with five males and five females, whose heights are in the 1.56 m–1.83 m range. The method applied in tuning *k* in [20] is similar to the approach used in [17] by optimization analysis based on experiment data, achieving minimum error performance for all data sets. The step length is in meter units:

(4) $${\text{step}}_{\text{length}}=\text{k}*\text{height}*\sqrt{{\text{step}}_{\text{frequency}}}$$

When detecting a valid step, the timestamp is recorded and the step frequency is calculated as the reciprocal of the time interval between two valid steps. The step frequency is updated every time a step is detected and the updated value is used to evaluate the length of the step.

For the initial step from stationary state to walking, the step length cannot be calculated according to Equation (4) as only one step timestamp is available. The step length is then estimated using the static model [31] where step length is proportional to height. *k_s_* is a constant and its value is set to 0.415 for male or 0.413 for female guided by [31]. The step length is in meter units.

(5) *step_length_* = k_s_*height

Since human step frequency has a minimum value of 0.5 Hz, the step frequency variable is set to zero when no valid step is detected for 2 s. Therefore, the step is considered as the initial step from stationary state to walking when the step frequency is found to be zero. Also, the step frequency is an indication of the pedestrian’s motion state. A non-zero step frequency indicates walking and a zero step frequency corresponds to standing still.

Step orientation is estimated utilizing the yaw data from the gyroscope. Yaw data in radians indicates the angle turned around the phone’s *z*-axis. When the smartphone is held in the hand, the yaw data directly reflect the orientation of walking and making a turn will have equivalent changes in the yaw data samples. After detecting a step, all yaw samples within the step interval are averaged to obtain the orientation of the step used in updating the position according to Equation (1). Since the yaw data samples are presented in range of (–π, +π], the 180^o^ ambiguity issue is caused when yaw data changes around ±π. In such a situation, the averaging of raw yaw data is performed according to the pseudo code presented in Algorithm 1 where *yaw_raw_* is the array of yaw data recorded during the step, *yaw_inter_* is an intermediate array holding temporary values of yaw data during processing, *average* is the operation to get the numerical average value of an array and *yaw_cali_* is the output of the calibrated average value of the array.

Algorithm 1 Averaging Yaw Data in 180^o^ Ambiguity
   Given an array of raw yaw data *yaw_raw_* with *p* samples   *for* *i* = 1: *p*      *if yaw_raw_*(*i*) < 0          *yaw_inter_*(*i*) = *yaw_raw_*(*i*) + 2*π       *else*           *yaw_inter_*(*i*) = *yaw_raw_*(*i*)      *end if*   *end for*   *if average*(*yaw_inter_*) > π      *yaw_cali_* = *average*(*yaw_inter_*)−2*π  *else*      *yaw_cali_* = *average*(*yaw_inter_*)  *end if*

### 3.3. SRP Adaptive Drift Calibration

In the proposed HILN system, the adaptive drift calibration (ADC) based on SRP interaction is a mechanism that aims at reinitializing position errors and calibrating sensor drift of PDR subsystem, enabling a drift-free system capable of providing long-term accurate positioning and tracking performance. Since the proposed PDR is step-based, drift in accelerometer has little influence on positioning accuracy and the main source of error is caused by the drift in gyroscope.

**Figure 4:**
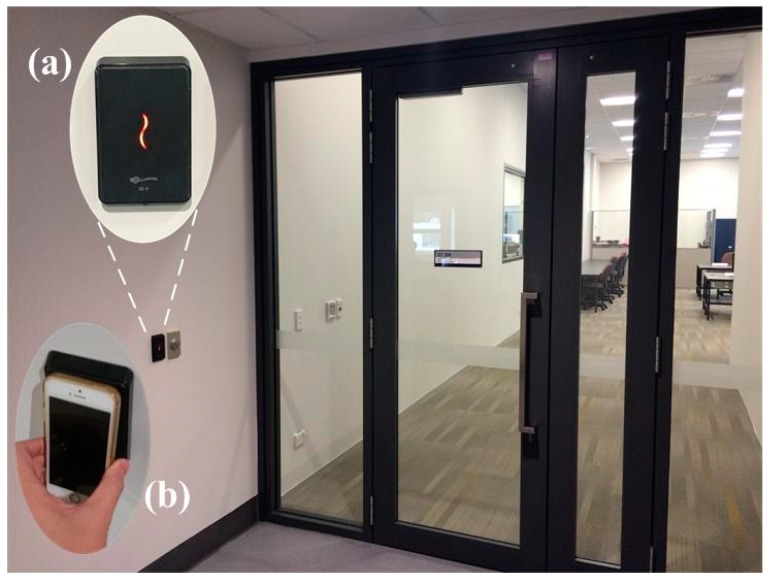
Access control system (**a**) Access card reader (**b**) Phone position against reader.

Figure 4 presents the elements of access control system used for integration of SRP based ADC scheme in the proposed HILN system. The figure shows the entrance of a laboratory in the experiment area. Access into the room is controlled by swiping access card against the reader as shown in Figure 4a. To integrate the ADC subsystem with the PDR subsystem implemented on smartphone, the access card is attached to the back of smartphone and used in the way as shown in Figure 4b to gain access to the room. A similar setting of SRP access card reader is also used inside the room for unlocking the door when leaving the lab. Additional information on this is provided in Section 4.

The access control system relies on RFID, which has a working range of about 5 cm, and in terms of performance is similar to NFC, commonly supported in modern smartphones. Thanks to the short-range characteristic of SRP signals, the exact position of the pedestrian can be established. When entering or leaving a room by swiping an access card against the reader, a drift calibration process is initiated and the pedestrian’s position is set to the entrance or exit of the room, hence effectively resetting any accumulated position errors. More specifically, the position of the pedestrian is set to be 0.5 m away from the location where the reader is installed (similar to the length of the arm) in the calibration.

Moreover, the gyroscope drift can also be corrected in the calibration process during SRP interaction. Since the step orientation is estimated solely using yaw data, only the yaw drift is corrected. When the smartphone is swiped against the reader as in Figure 4b, the actual orientation of the smartphone can be determined with knowledge of the placement of the reader. By comparing the difference between the actual orientation and the measurement data from gyroscope, the gyroscope drift can be determined by Equation (6), where *yaw_drift_*, *yaw_measure_*, *yaw_true_* denote the yaw drift, gyroscope measurement and actual yaw orientation during the SRP interaction, respectively. Afterwards, the drift-free gyroscope data *yaw_dr-free_* can be obtained by removing the sensor drift from measurement data as expressed in Equation (7):

(6) *yaw_drift_* = *yaw_measure_* – *yaw_true_*

(7) *yaw_dr-free_* = *yaw_measure_* – *yaw_drift_*

**Figure 5:**
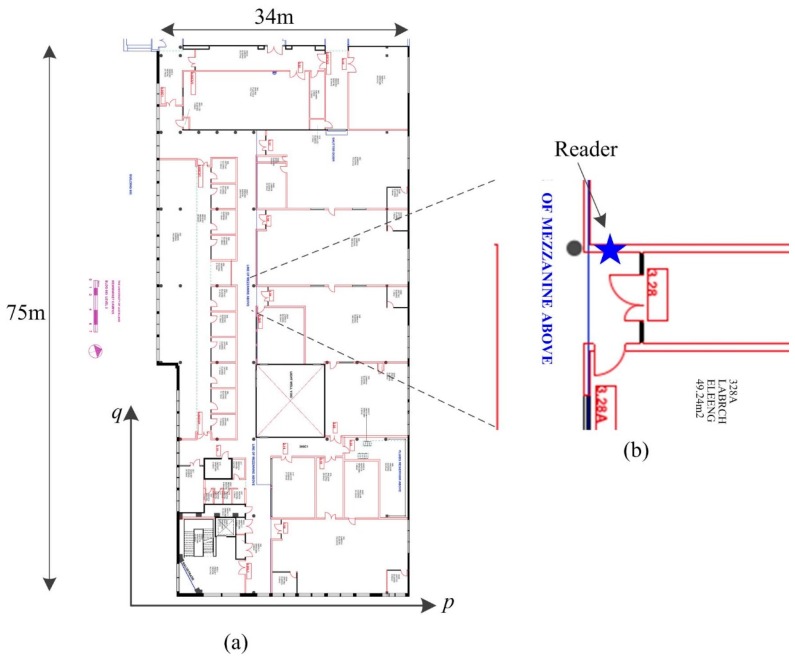
(**a**) Map of the test indoor environment (**b**) Example of access control system.

Figure 5a is the layout (map) of an indoor environment of Level 3, Building 903, Newmarket Campus, at the University of Auckland, where the experiments were conducted. Figure 5b is the detailed view of a laboratory entrance. An example is given as follows regarding the yaw drift calibration process based on the knowledge of access card reader placement. In the coordinate system defined in Figure 5a, if the yaw sample of gyroscope is 0 *rad* when the pedestrian is walking along the positive *p*-axis with the smartphone held in hand, then the yaw samples will be *π*/2 *rad* and –*π*/2 *rad* ideally when walking along the positive and negative *q*-axis. An access card reader, denoted by a blue star, is installed upright on the walls as shown in Figure 5b (also refer to Figure 4). When swiping a smartphone against the reader as in Figure 4b, the yaw orientation of the phone should ideally be *π*/2, indicating the device is facing towards the positive *q*-axis. Therefore, the sensor drift can be calculated using Equation (6) by comparing the yaw data measurement with the ground truth orientation.

In addition, by integrating PDR with the access control system upon entering or leaving a room, the room position information (RPI), describing whether the pedestrian is in the corridor or in a specific room, can be accurately obtained. The interaction with the access control system corresponds to a change in the RPI. For example, an interaction with the reader at the room entrance indicates the RPI changes from corridor to the specific room, whereas the interaction made at the room exit corresponds to the transition from the specific room into the corridor in RPI.

By integrating the PDR system with access control using the SRP signal, a drift-free positioning and tracking performance is achieved. The position error is regularly corrected when entering or leaving a room and the gyroscope drift is corrected when the phone is swiped against a reader with a known position and orientation.

### 3.4. Particle Filter Map Matching

A novel simplified PF map matching algorithm designed specifically for pedestrian navigation system with a robust lost track recovery mechanism is presented in this section to further enhance the localization and tracking accuracy of the HILN system.

PF [33] is a powerful Monte Carlo-based method addressing dynamic Bayesian system state estimation problems and widely applied in tracking and navigation [34]. The probability density function (PDF) of system states based on a series of observations is recursively approximated by a group of particles with sampling, weight calculation and resampling processes. The resampling process [35] is introduced to solve the performance degeneracy problem by discarding particles with negligible weights and replicating particles with dominating weights.

In the above presented step-based PDR system with two-dimensional coordinates, the system state evolving with steps is presented in Equation (1). In the state transition, additive noise is observed in both step length *L_s_* and orientation *θ* due to errors in step length modeling and sensor drift, as well as bias introduced by the pedestrian in the gyroscope, respectively. Given the fact that travelled distance is accurately estimated in the literature with less than 5% median error of the total travelled distance under different kinds of step length models [17,20], no additive noise is modeled in step length to simplify the algorithm as the system is targeted to achieve real-time performance on a standard smartphone device, so the system state transition functions are modeled as in Equations (8) and (9), which are the state propagation equations used in the PF. *θ_m_* denotes the step orientation obtained directly from gyroscope measurement, where the true orientation *θ_true_* is corrupted with noise of *θ_drift_* and *θ_bias_*. *θ_drift_* represents the sensor drift in the gyroscope and *θ_bias_* is the bias caused by the pedestrian holding the phone non-ideally while pointing in the walking direction. In traditional PF, a measurement model is also required and the weight of each propagated particle, $w_{t}^{i}$, is evaluated utilizing the measurement value, *z_t_*, and importance desity, $g(S_{t}^{i}|S_{t-1}^{i},z_{t})$ according to Equation (10), where *t*, *i* and *S* are time-step index, particle index and the state vector, respectively. In the simplified PF map matching algoritm in this paper, the measurement model is simplified in the form of the constraints imposed by the indoor map and a binary weight scheme [18] is used in weight calculation. It reduces the computational complexity of traditional PF by replacing complex computations in weight calculation step to binary mapping of particles, *i.e.*, the weight of propagated particles within valid range of the map equals to 1 while particles outside the valid area have the weight of 0:

(8) $$\left[\begin{array}{c} {\text{x}}_{\text{s}+1}\\ {\text{y}}_{\text{s}+1} \end{array}\right]=\;\left[\begin{array}{c} {\text{x}}_{\text{s}}\\ {\text{y}}_{\text{s}} \end{array}\right]+{\text{L}}_{\text{s}}*\left[\begin{array}{c} {\text{sin}\theta }_{\text{m}}\\ {\text{cos}\theta }_{\text{m}} \end{array}\right]$$

(9) $$\theta_{\text{m}}=\theta_{\text{true}}+\theta_{\text{drift}}+\theta_{\text{bias}}$$

(10) $${\text{w}}_{t}^{i}\;\propto w_{t-1}^{i}\frac{p(z_{t}|S_{t}^{i})p(S_{t}^{i}|S_{t-1}^{i})}{g(S_{t}^{i}|S_{t-1}^{i},z_{t})}$$

The goal of the PF map matching algorithm is to constrain the position within the valid area of indoor environment when using noisy gyroscope measurements. In the proposed HILN system, the accurate RPI of the pedestrian is provided by the SRP ADC subsystem, so this information is also introduced in addition to the map constraint for better performance. The map and RPI help to restrict the position and walking track of the pedestrian to be within the range of corridors or rooms.

**Figure 6:**
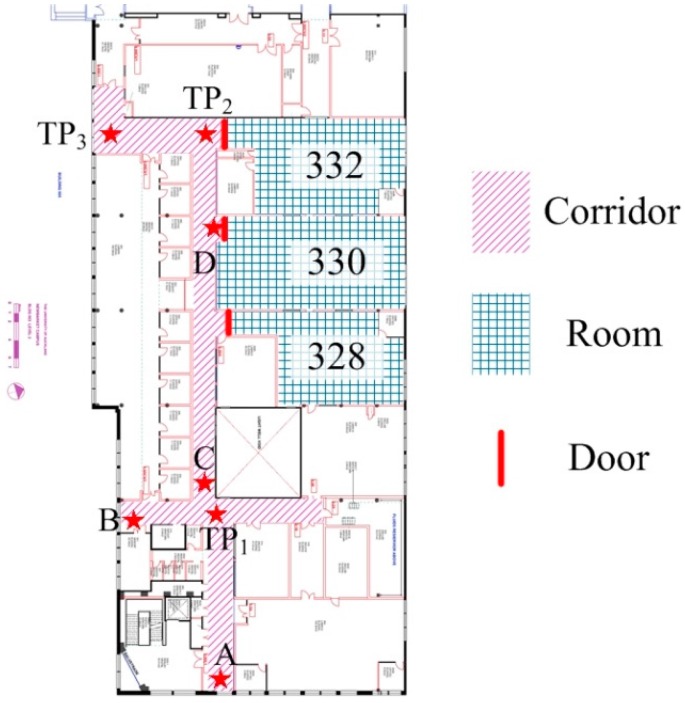
Illustration of different zones.

Figure 6 presents the way in which valid map area is divided into different zones based on the RPI with three rooms numbered 328, 330 and 332 as examples. In the map matching algorithm of HILN system, the position is restricted to a specific zone according to the RPI. For instance, only the corridor zone is considered as the valid area when the pedestrian is in the corridor.

The pseudo code for PF map matching using *N* particles is presented in Algorithm 2 as well as the symbol definitions. During initialization, the position and all particles are set to a known starting position. Regarding the experiment area presented in Figure 6, *MAP* is the set of all valid positions in the area, *POS_RPI_* is the set of all possible RPI {*Corridor*, *Room_328_*, *Room_330_*, *Room_332_*} and *TP* denotes the set of all turning points {*TP_1_*, *TP_2_*, *TP_3_*}, which are the center points of a turning zone.

After detecting a valid step, the step length and orientation is obtained from the PDR subsystem. Also, current RPI is used to select a subset from *MAP*, containing only locations in the corresponding RPI zone. Then, all particles are propagated with random noise added to the step orientation. A binary weight is assigned to every particle at the same time. If not all weights of particles are zero, the position of the pedestrian is updated as the weighted average of the particle set. Since particles with non-zero weights are in the valid map area, the weighted average of the particle set will always fall within the constraints of the map. Afterwards, particles are resampled with standard systematic resampling [35] before the next iteration. Particles outside the valid map area are discarded and surviving particles are replicated, maintaining the total number of particles at *N*.

Since the weight is binary, it is possible that all particle weights are zero. This situation is defined as a lost track where all propagated particles are outside the range of current valid zone. In the proposed algorithm, the issue of lost track is addressed by a novel lost track recovery (LTR) mechanism based on current position and RPI.

**Table:** 

**Algorithm 2** Particle Filter Map Matching for Pedestrian Tracking
1. **Initialization**: 2. Initial position $P_{0}={\left[x_{init},\;y_{init}\right]}^{T}$, initialize particle set *Particle_0_*, $p_{0}^{i}={\left[x_{init},\;y_{init}\right]}^{T}\;\left(1\leq i\leq N\right)$ 3. *MAP* is a set containing all valid positions in a map 4. *POS_RPI_* is a set containing all RPI {*Corridor*, *Room_r_* (*r* = valid room number)} 5. *TP* is a set containing all turning point positions in corridor zone with a total number of *M* 6. **at step index *s+1*** (s≥0): 7. get the estimated step length *L_s_* and orientation θ from PDR subsystem 8. get current RPI $pos_{rpi}\in \;POS_{RPI}$, the current valid position set $valid_{pos}\in \;MAP$ is selected based on *pos_rpi_* 9. propagate particle set *Particle_s_* to *Particle_s+1_*, assign weight to each propagated particle 10. ***for*** *i* = 1 to *N* 11. draw a random number *rand* from *N*(*0, σ*^2^), $\theta_{m}^{i}$ = θ + *rand* 12. propagate particles $p_{s}^{i}$ to $p_{s+1}^{i}$ according to Equation (8) 13. assign particle weight $w_{s+1}^{i}$ 14. $w_{s+1}^{i}=\{\begin{array}{c} 0,\;if\;p_{s+1}^{i}\;\;\notin \;valid_{pos}\\ 1,\;if\;p_{s+1}^{i}\;\in \;valid_{pos} \end{array}$ 15. ***end for*** 16. ***if*** all particle weights are zero 17. ***if*** $pos_{rpi}$ equals *Corridor* and current position *P_s_* is not in zones where lost track is allowed^*^ 18. go to **Lost Track Recovery** 19. ***else*** 20. *P_s+1_* = *P_s_*, *Particle_s+1_* = *Particle_s_* 21. ***end if*** 22. ***else*** 23. $P_{s+1}=\overset{N}{\underset{i=1}{\sum }}p_{s+1}^{i}w_{s+1}^{i}/N$, Resample particle set *Particle_s+1_* using Systematic Resampling 24. ***end if*** 25. ***end of processing* at step index *s+1*** 26. 27. **Lost Track Recovery** 28. **Start:** 29. select the turning point *TP_k_* ∈ TP (1≤k≤M), having the minimum distance to *P_s_* in *TP* 30. ***if** TP_k_* is not unique 31. *P_s_* = *P_s-1_*, go to **Start** 32. ***else*** 33. *P_s+1_* = *TP_k_*, $p_{s+1}^{i}=TP_{k},\;\left(1\leq i\leq N\right)$ 34. ***end if***

^*^ rooms, the boundary of map and zones near room entrance in corridor is considered as zones where lost track is allowed.

It is observed that a lost track happens when the pedestrian is making a turn where the particles may have not reached or have passed the turning zone to make the corresponding turn and satisfying the map constraints at the same time. Therefore, the position and particles are corrected to the turning point having the minimum distance to current position of the pedestrian during LTR. If the turning point is not unique, *i.e.*, more than one turning point have the same minimum distance to current position is possible, the position history of the pedestrian is used until the unique turning point requirement is satisfied. As shown in the illustration in Figure 6, the pedestrian walks from point *A* to point *B* with a left turn. When the person reaches the turn, the position might be erroneously estimated to point *C* due to overestimated step length. Thus all particles are in proximity to point *C*. After turning left, all particles will turn invalid as the path is blocked by the wall presented in the map. By introducing LTR mechanism, the position and particles are corrected to the nearest turning point *TP_1_*. Thus, the path is recovered and continues stretching left along the corridor after the turn.

To enhance the robustness of the LTR mechanism, lost track is allowed in rooms and certain zones of the corridor including map boundaries and the proximity of every room entrance. When a lost track is identified in these zones, both the position and particles remain unchanged, without any update.

Also referring to Figure 6 in the path from point *A* to *B*, after the turning point *TP_1_*, all particles might go out of the map boundary when the pedestrian reaches point *B* due to overestimated step length. Moreover, when approaching the room entrance in corridor, point *D* for example, it is possible that all particles become invalid as the room entrance is also the boundary of the corridor. In these situations, the position and particles should remain stable.

Meanwhile, rooms are considered as free spaces. Lost tracks are allowed as long as the position of the pedestrian is restricted within the room. The RPI provided by the SRP subsystem is an accurate indication of the pedestrian’s location.

By employing the PF map matching algorithm in pedestrian tracking, map constraints are introduced to improve the positioning and tracking accuracy. The LTR mechanism contributes to enhancing the robustness of the algorithm against lost track issues when making turns.

## 4. Evaluation & Discussion

This section presents the evaluations of HILN system in pedestrian localization and navigation with experimental setup, short-term and long-term walking experiment and discussions, respectively.

### 4.1. Experimental Setup

**Figure 7:**
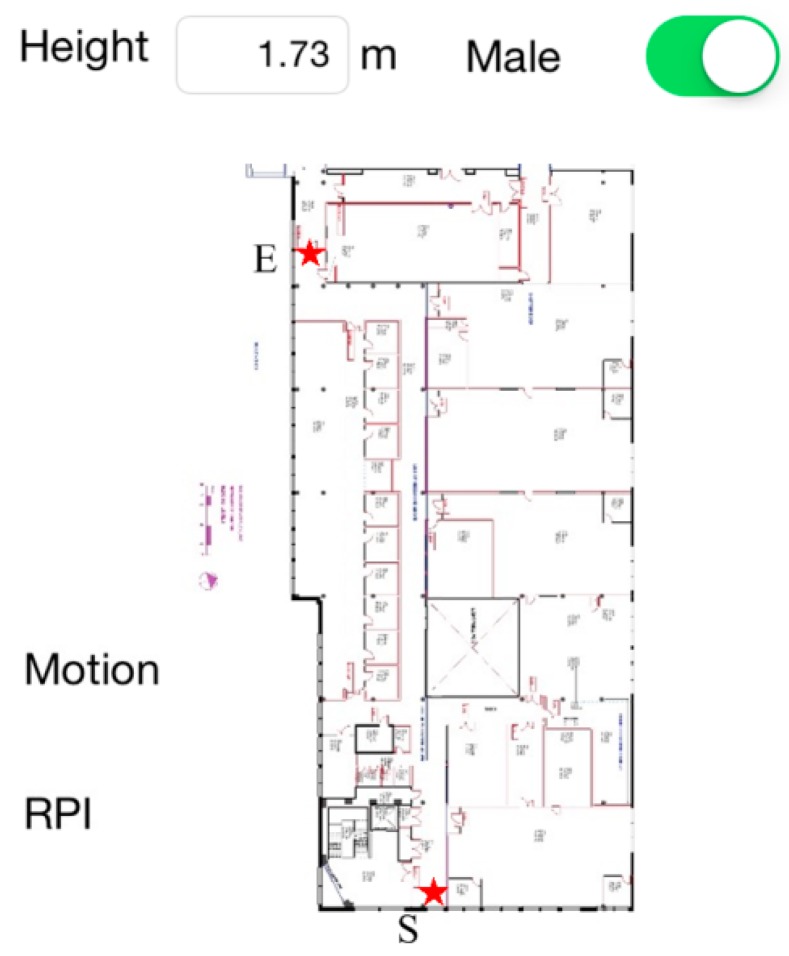
Application screenshot.

The proposed HILN system is implemented on an iPhone 5s sampling accelerometer and gyroscope data at 50 Hz. The sensor data and the sampling timestamps are also recorded for more comprehensive offline analyses. Figure 7 is a screenshot of the developed application run with the map layout of the area used in experiments. The text box and switch above the map can be used to adjust the height and gender of the tracking target, which is set to 1.73 m and male by default, as the information is related to the step length model in Equations (4) and (5). The two labels to the left of the map are used to indicate the motion state, *i.e.*, walking or standing, and RPI, *i.e.*, in corridor or in a specific room, of the pedestrian. In implementing the SRP adaptive drift calibration feature, the access card is attached to the back of the smartphone and swiped against the reader as in Figure 4b. The calibration process is emulated by an HTTP message, containing position error correction and gyroscope yaw drift calibration information, sent to the application from an external computer when user interaction between the smartphone and the reader is performed. The point *S* labeled with a star indicates the initial position of the pedestrian to be tracked while point *E* denoted the upper boundary of corridor in the experiment area. During walking, the smartphone is carried in hand as in Figure 2b, which is a reasonable position commonly found in the literature [16,18,20,21,22,23,26] when people navigate themselves in indoor environment. Also, it is more convenient for the pedestrian to hold the device in hand as the phone is regularly used to interact with the access control reader when entering or leaving rooms.

For the PF algorithm, the number of particles used in pedestrian tracking varies from 100 [15] to 200 [23] in the literature. In order to ensure low computational complexity in implementing the simplified PF map matching algorithm, a total number of 100 particles are used. The variance of the normal distribution *N*(0, *σ^2^*) used in modeling additive noise in step orientation, *i.e.*, *σ^2^*, is set as 0.22, corresponding to a 99.7% probability that the random number is within the range of [−0.6 *rad*, 0.6 *rad*] (0.6 *rad* = 34.4°). The value is set based on observations that yaw data drift is within this range. Turning points defined for LTR mechanism in the corridor zone are the same as presented in Figure 6.

### 4.2. Short-Term Walking Experiments

**Figure 8:**
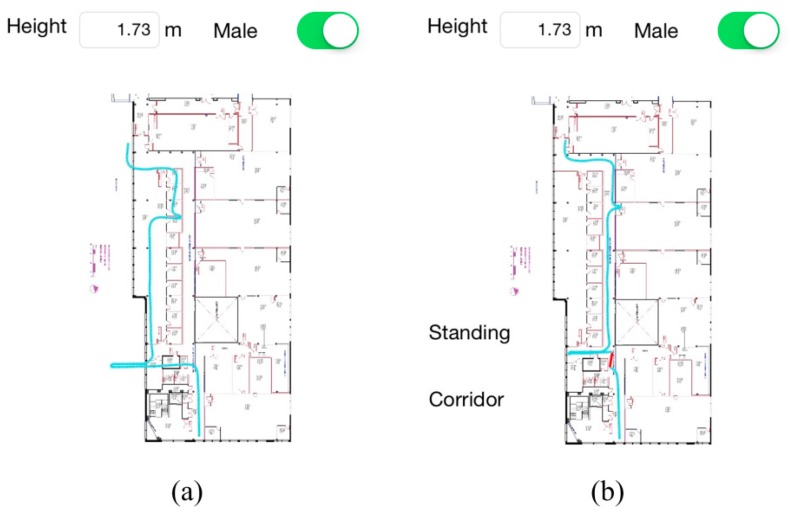
First short-term walking experiment result: (**a**) Path generated offline with the PDR standalone approach; (**b**) Path generated real-time by HILN system.

Two short-term walking experiments are conducted. The route in the first experiment is in corridor and focuses on the effectiveness of the LTR mechanism. Figure 8 presents the tracking results where the path generated in real-time on the smartphone by HILN system is illustrated in Figure 8b and the path generated using the same set of data offline using PDR standalone approach is depicted in Figure 8a. In the experiment, the actual starting point of the walk is a few steps ahead the point *S* as in Figure 7 in order to cause lost track in a shorter distance, so when the subject reaches the turning zone, the path drawn by the application has not reached the area yet. By turning left, the path is blocked by the wall and all particles become invalid. As can be seen in Figure 8b, the path is immediately recovered to the turning point of *TP_1_*, drawn with a red line, and continues to track the subject accurately. After getting to the leftmost point of the map, a few extra steps are made deliberately at the same location to force all particles to be outside the boundary of the map. As lost track is allowed at a map boundary, the particles are not propagated anymore and the position of the pedestrian remains stationary. Then the subject turns back and keeps walking with a left turn along corridor towards the entrance of Room 330, which is accurately tracked as seen from the figure. After arriving at the entrance, a few extra steps are also made at the same spot to force all particles to be outside the boundary of the corridor (*i.e.*, getting into Room 330). Afterwards, the subject turns back and continues walking along corridor to point *E*. The tracking result presented proves that particles and the pedestrian’s position are not reset when a lost track is identified in the proximity of a room entrance. The whole path in the experiment is also accurately plotted by the application.

The path generated offline with the PDR standalone approach using the same set of data, presented in Figure 8a, is obviously influenced by behaviors during the experiment. Since the real starting point is a few steps ahead the point *S*, error is observed in the path after the first left turn. Also, the path stretches beyond the left-side map boundary due to the extra steps made on the same location. The influence of those faked steps is observed near the entrance of Room 330 as well. Seen from the overall path, the position errors continue to affect the tracking performance throughout the experiment once introduced.

The route taken in the second experiment starts from point *S* towards point *E* along the corridor. Then the subject enters Room 332 and returns to point *S*. The total length of the path is 202.8 m and the actual step count is 273. The tracking performance of the HILN system is summarized in Table 2. The step detection rate and estimated total travelled distance (TTD) achieved over 99% accuracy. With the help of the SRP ADC subsystem, the position error and gyroscope yaw data drift are corrected when the subject enters and leaves Room 332. The position error and gyroscope drift calibrated are 1.19 m and 0.78 m, 0.011 *rad* and 0.033 *rad*, respectively. As the subject returns to the starting point when the walk finishes, the final position error is measured as 1.51 m, 0.74% over TTD.

**Table 2:** Tranking performance of the second short-termexperiment.

**Actual Step Count**		273
**Detected Step Count/Accuracy**		271/99.27%
**Travelled Distance (m)**		202.8
**Estimated Total Travelled Distance (m)/Accuracy**		202.1/99.65%
**Position Error Corrected (m)**	Entering Room 332	1.19
	Leaving Room 332	0.78
**Gyroscope Yaw Drift Corrected (rad/deg)**	Entering Room 332	0.011/0.63^o^
	Leaving Room 332	0.033/1.89^o^
**Final Position Error ε (m)**		1.51
**ε/TTD**		0.74%

**Figure 9:**
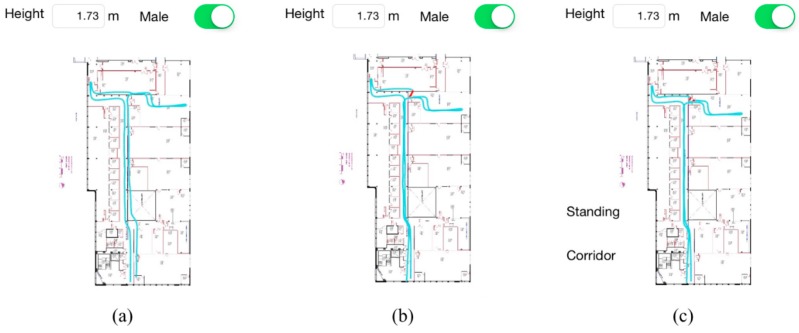
Tracking path of the second short-term experiment (**a**) PDR standalone (**b**) PDR + SRP ADC (**c**) HILN.

The tracking path of the experiment is presented in Figure 9 where the red path denotes the position correction by the SRP ADC subsystem. Figure 9c is the real-time generated path during the walk presented on the smartphone. Figure 9a is the path using PDR standalone approach and Figure 9b illustrates the path combining PDR and the SRP ADC subsystem.

When using the PDR only approach, the position error accumulates over time due to the inaccuracy in step length and gyroscope drift. After combining the SRP ADC subsystem, the position error and yaw data drift are corrected, providing better tracking performance, but the path still occasionally falls into invalid zones of the map. The tracking path of the HILN system enhances the performance further by integrating constraints imposed by the map information. The final position errors of the three paths are 2.4 m, 1.76 m and 1.51 m, respectively. In addition, the corrected position errors in Figure 9b when entering and leaving Room 332 are 2.78 m and 0.13 m, respectively. HILN system not only improves the final position accuracy, but also the positioning and tracking performance throughout the path, which is observed in Figure 9.

### 4.3. Long-Term Walking Experiment

A long-term walking experiment is carried out to verify the drift-free accurate tracking of the HILN system. Starting from point *S*, the subject makes a 998 s (16.6 min) walk exploring all the corridor area, visiting Room 328 and Room 332 many times and returning to the initial position of point *S* when the walk finishes. A total number of 1486 steps are taken in the route, while the developed application successfully detected 1449 steps with 97.51% accuracy. The total travelled distance is 1062.21 m reported by the application, achieving 97.99% accuracy as the ground truth distance is 1083.95 m. The quantified tracking results are presented in Table 3. During the experiment, the subject enters Room 328 and Room 332 both four times, so a total number of 16 calibrations are processed in the SRP ADC subsystem. The maximum, minimum and average values of position error corrected when entering or exiting rooms are 2.76 m, 0.2 m and 1.23 m, respectively. Meanwhile, the maximum, minimum and average values of yaw data drift calibrated are 0.402 *rad*/23.04°, 0.034 *rad*/1.95° and 0.202 *rad*/11.58°, respectively. When the walk finishes, the final position error is 1.36 m, 0.13% over TTD.

**Table 3:** Tracking performance of the long-term experiment.

**Actual Step Count**		1486			
**Detected Step Count/Accuracy**		1449/97.51%			
**Total Travelled Distance (m)**		1083.95			
**Estimated Total Travelled Distance (m)/Accuracy**		1062.21/97.99%			
**Position Error Corrected (m)**	Entering Room 328	2.76	0.57	1.39	0.57
	Leaving Room 328	0.95	0.85	0.20	0.70
	Entering Room 332	1.55	1.34	1.22	1.75
	Leaving Room 332	1.68	1.56	1.29	1.31
**Gyroscope Yaw Drift Calibrated (rad/deg)**	Entering Room 328	0.068/3.90^o^	0.210/12.04^o^	0.238/13.64^o^	0.345/19.78^o^
	Leaving Room 328	0.039/2.24^o^	0.034/1.95^o^	0.117/6.71^o^	0.242/13.87^o^
	Entering Room 332	0.137/7.85^o^	0.224/12.84^o^	0.214/12.27^o^	0.402/23.04^o^
	Leaving Room 332	0.071/4.07^o^	0.251/14.39^o^	0.235/13.47^o^	0.402/23.04^o^
**Final Position Error ε (m)**		1.36			
**ε/TTD**		0.13%			

**Figure 10:**
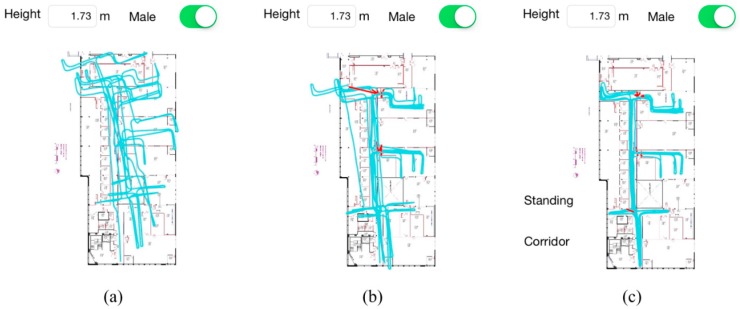
Tracking path of the long-term experiment (**a**) PDR standalone (**b**) PDR + SRP ADC (**c**) HILN.

Similarly, the tracking paths using PDR only, PDR + SRP ADC and the real-time plotted path by HILN system are presented in Figure 10 using the same set of data during the experiment. Red lines in the path represent the position correction in SRP ADC subsystem and LTR in PF map matching algorithm. The final position errors of the three paths are 22.08 m, 1.84 m and 1.36 m, respectively. By using four access points (two per room) in the experiment area, the final position error is significantly reduced from 22.08 m to 1.36 m. With more access points utilized, the overall performance will be further improved since the errors in PDR system will be more frequently corrected.

As can be seen from Figure 10a, the path obviously deviates from the actual one due to gyroscope drift when only PDR is used. With the help of the SRP ADC subsystem, by regularly correcting position errors and yaw data drift, the path fits better to the real route travelled by the pedestrian. Nevertheless, the gyroscope drift between two calibration processes leads to inaccurate positioning and tracking results. For instance, the leftmost part of the path in Figure 10b deviates significantly from the corridor because of yaw data drift. The maximum, minimum and average position errors corrected by SRP ADC subsystem in the result of Figure 10b are 11.04 m, 0.36 m and 2.33 m, respectively. With further enhancement introduced by the PF map matching algorithm, an accurate positioning and tracking performance is observed along the whole experiment path plotted by HILN system. In the experiment, lost track happened once and the timestamp of incidence during the 998 s walk was 791.5 s as indicated in Figure 10c near turning point *TP_1_*. The path is successfully recovered to continue tracking of the subject. The long-term experiment demonstrates the advantages of the HILN system to provide robust and drift-free pedestrian navigation independent of the travelled distance.

**Figure 11:**
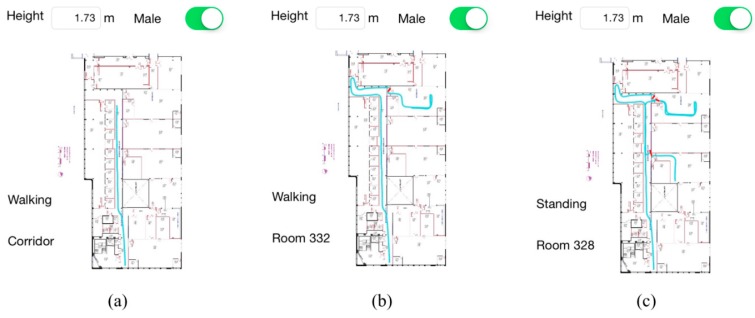
Intermediate tracking results during the long-term experiment (**a**) Walking in corridor (**b**) Walking in room 332 (**c**) Standing in room 328.

Figure 11 shows a collection of intermediate screenshots of tracking result in the first lap of the route. The two labels to the left of the map correctly show the motion state and RPI of the subject during the whole walking period and three examples illustrated in Figure 11a–c correspond to walking in corridor, walking in Room 332 and standing in Room 328, respectively.

**Figure 12:**
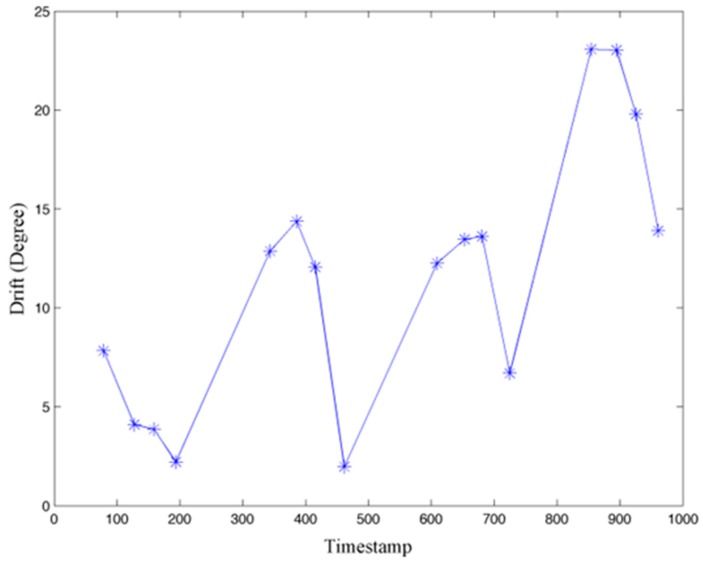
Gyroscope drift in yaw measurement.

Figure 12 presents the calculated gyroscope drift in yaw measurement during user interaction in the 998 s experiment with increasing timestamp. It is observed that drift in the yaw measure does not always become larger over time as the minimum drift appears at the 8th time instance, 461 s, when leaving Room 328. Although the drift is always a positive value in this experiment, negative values appear in other experiments with similar routes.

### 4.4. Discussions

As the most significant feature of HILN system is the capability to offer drift-free accurate positioning and tracking performance, the long-term experiment result is firstly compared to [14,27] with comparable travelled distance. The final position error with respect to TTD reported in [14] is 1.06%, 0.37%, 0.22% and 0.15% when the number of ramps per 125 m is 0, 1, 2 and 3, respectively. The PDR-only result in this paper of 22.08 m, 2.04% over TTD, is not as good as the one in [14] when no ramp is used for drift correction, but the path in [14] is a regular square path, less complex than the one presented in this paper with an irregular shape. When drift correction methods are applied, the HILN system outperforms the accuracy reported in [14] with only 0.13% error over TTD. For [27], ε/TTD is 0.15%, 0.45%, 0.25% and 0.1% for different path length of 600 m, 550 m, 520 m and 1000 m. While the performance of HILN is marginally higher than the 0.1% final position error, the system in [27] requires 71 RFID tags to be pre-installed in the building with an area of 40 m × 60 m (2400 m^2^). In the HILN system, comparable performance is achieved by using only four SRP nodes in the experiment area of 75 m × 34 m (2550 m^2^). Regarding the correction methods, ramp detection [14] relies on the structural features of the building and RFID ranging [27] requires additional infrastructure support, incurring significant cost, while the access control system used in this paper is more commonly employed in buildings nowadays and can be readily installed as well.

Moreover, the experiment route covers both rooms and corridor in indoor environment and HILN system is able to provide accurate RPI at the same time, whereas experiments presented in [14] are for corridor-only scenarios. Other works [13,18,19,20,21,23,24] are limited to corridor scenarios, as well. Since people walking in indoor environments are typically moving from one room to another via corridors, it is necessary to cover both parts of the building for a PDR system. The RPI also enhances the advantages of the HILN system over traditional PDR systems with PF map matching. In [15], the measurement model of PF is also simplified into the form of map constraints. Without the RPI, the binary weight of particles is assigned based on whether the propagation path of particles crosses wall or not since rooms are also valid locations of pedestrians [15]. Nevertheless, the evaluation of the system is only conducted in a corridor environment, while in the HILN system, the RPI is used to provide more detailed map constraints for the PDR system. The process of weight evaluation can also be simplified by checking whether the position of particles is in valid area or not.

The accuracy of step detection and estimation of TTD is then compared to previous works (also refer to Table 1). In terms of step detection rate, the accuracy of 99.27% reported in the short-term experiment which covers 202.8 m is comparable with the close to 100% accuracy in [16] and better than the accuracy of 98% reported in [17]. The step detection rate of 97.51% in the long-term experiment is not as good as that in [16,17]. As for estimation of TTD, the accuracy of both short-term and long-term result in the HILN system outperforms that of [19]. Meanwhile, performance is comparable with other works [13,16,20], all offering performance of less than 3% error.

Despite the fact that the accuracy of the HILN system is marginally lower compared to some of the previous works, the differences in experiment scenarios, *i.e.*, the TTD and shape of the path, should be considered. The TTDs in [13,15,16,17,18,19,20,21,22,23,24,25,26,28] cover from a few meters to a maximum of 600 m while the route presented in this paper has a total length of over one kilometer. Besides, the experiment in [16] is taken on a 400 m track in an outdoor sports field, whereas several independent walks in a straight path are conducted in [17,19] and the length of the path is 32 m in [17] and less than 7 m in [19], respectively. Paths of square shape are found in [13,14,15,20,21,23,24,25,26,27,28]. The path travelled as presented in this paper is much more complex and realistic for indoor scenarios with frequent stops and sharp 180° turns, which are not considered in previous works [13,14,15,16,17,18,19,20,21,22,23,24,25,26,27,28]. It is observed that the main reason for position errors in the proposed HILN system is undetected steps since the drift in yaw data is regularly corrected when entering/leaving a room and the position is constrained by the building map. The initial steps from stationary to walking state are missed more likely since the magnitude in vertical acceleration is relatively small. In the experiment, the subject has frequent transitions between stationary and walking state when entering or leaving the rooms. The transitions are also observed in sharp 180° turns when the subject reached the boundary of the map or returned to the exit after exploring the room, causing undetected steps. In addition, it is observed that the yaw data drift of gyroscope increases more drastically in sharp 180° turns.

**Figure 13:**
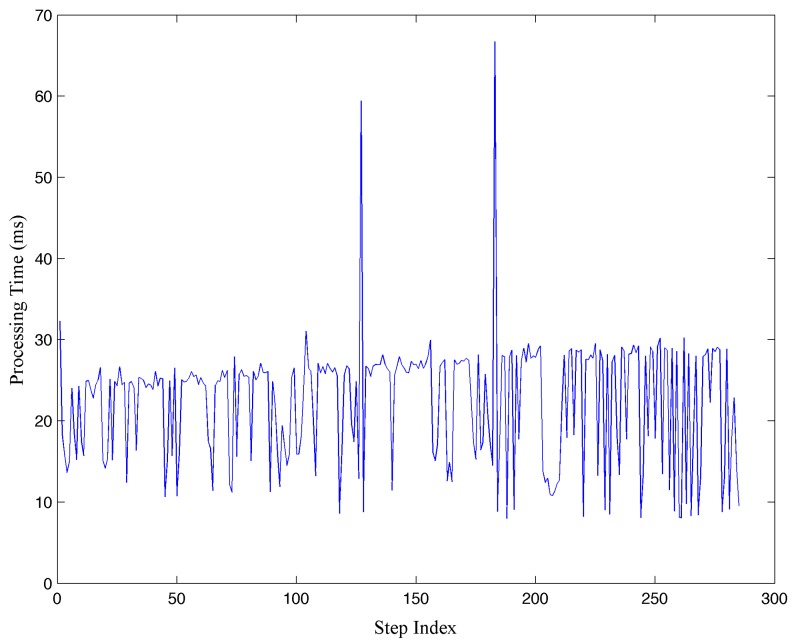
Example processing time of individual iterations.

Furthermore, the processing time of individual iterations when steps are detected during the short-term experiment is presented in Figure 13. The processing time of long-term experiment is similar since the processing flow is the same. After detecting a step, the processing time includes step length and orientation estimation, position update through a PF map matching algorithm with 100 particles and graphical processing updating the path of the pedestrian on the screen. Also, the smartphone is used normally and all the processing is scheduled by the operating system. As illustrated in the figure, the maximum processing time of one iteration is 66.7 ms, while most of the processing takes between 10 ms and 30 ms, with an average value of 22.5 ms. In the experiment, the subject walks at a step frequency of less than 2 Hz. Therefore, a time window of more than 500 ms is allowed for the processing of a valid step. Moreover, a minimum time window of 200 ms is available since the maximum step frequency of a pedestrian is 5 Hz. With maximum processing time of 66.7 ms, the HILN system achieves real-time positioning and tracking performance by using a standard smartphone.

In order to address the advantage of the simplified PF map matching algorithm over standard PF, the computation complexity is investigated offline. The simplification of PF mainly reduces the computations in weight calculation of particles. A Matlab program implementing the whole pedestrian tracking system with both simplified PF and traditional PF (the required measurement data is set as a constant since the simulation is only used to study the computation complexity) algorithm is used. With the same set of sensor data obtained in the long-term experiment, the average processing time consumed by a PF iteration when a valid step is detected is 0.42 ms for simplified PF and 0.72 ms for traditional PF, respectively, on a MacBook Pro host machine with 2.2 GHz Intel Core i7. Therefore, a 42% reduction in computation complexity is achieved. While regarding the localization, it is shown in previous works [22,23,24], where hybrid systems fusing PDR and indoor RSSI information are developed using a standard PF algorithm and the average localization error can be up to 5.46 m [23] and 5.22 m [24]. In [22], the average localization error is 0.88 m but experiments are only conducted along a 90 m path. Also, additional dedicated infrastructure is required in [22,23,24]. The proposed HILN system achieves drift-free, satisfactory localization performance with minimum computational and infrastructure cost.

## 5. Conclusions and Future Work

A hybrid indoor localization and navigation (HILN) system for pedestrians is proposed in this paper. The system integrates a traditional PDR system with SRP adaptive drift calibration at access control points and a particle filter map matching algorithm. The information derived from SRP subsystem enables PDR to offer drift-free tracking and by taking advantage of existing access control systems, it offers a natural way to integrate user interactions at no additional cost. The map constraints, introduced by a simplified particle filter with LTR mechanism, further enhance the accuracy and keep the computation complexity acceptable for real-time processing on mobile devices with limited resources. The long-term experiment verified the robust and reliable tracking of the proposed system with 0.13% final position error with respect to a total travelled distance of over one kilometer. Moreover, the accurate tracking performance is independent of travelled distance and the system covers both corridor and rooms of indoor environments, offering a more practical way for pedestrian navigation.

Future work will focus on extending the system to support different ways of carrying the smartphone during walking. Also, a more comprehensive system will be studied combining both outdoor and indoor environments to provide seamless tracking of the pedestrians.

## References

[1] Du Y., Yang D., Xiu C. (2015). A Novel Method for Constructing a WIFI Positioning System with Efficient Manpower. Sensors.

[2] Au A.W.S., Chen F., Valaee S., Reyes S., Sorour S., Markowitz S.N., Gold D., Gordon K., Eizenman M. (2013). Indoor Tracking and Navigation Using Received Signal Strength and Compressive Sensing on a Mobile Device. IEEE Trans. Mob. Comput..

[3] Zhang P., Zhao Q., Li Y., Niu X., Zhuang Y., Liu J. (2015). Collaborative WiFi Fingerprinting Using Sensor-Based Navigation on Smartphones. Sensors.

[4] Shikur B.Y., Weber T. TDOA/AOD/AOA localization in NLOS environments. Proceedings of the IEEE International Conference on Acoustics, Speech and Signal Processing.

[5] Mario M., Pedro J.M., Carlos D.K. (2012). Using Bluetooth to implement a pervasive indoor positioning system with minimal requirements at the application level. Mob. Inf. Syst..

[6] Lim A., Zhang K. (2006). A Robust RFID-Based Method for Precise Indoor Positioning. Advances in Applied Artificial Intelligence.

[7] Hsu C.-C., Chen J.-H. (2011). A Novel Sensor-Assisted RFID-Based Indoor Tracking System for the Elderly Living Alone. Sensors.

[8] Na J. (2006). The Blind Interactive Guide System Using RFID-Based Indoor Positioning System. Computers Helping People with Special Needs.

[9] Ozdenizci B., Kerem O., Coskun V., Aydin M.N. Development of an Indoor Navigation System Using NFC Technology. Proceedings of the Fourth International Conference on Information and Computing.

[10] Puertolas-Montañez J.A., Mendoza-Rodriguez A., Sanz-Prieto I. (2013). Smart Indoor Positioning/ Location and Navigation: A Lightweight Approach. Int. J. Interact. Multimed. Artif. Intel..

[11] Akeila E., Salcic Z., Swain A. (2014). Reducing low-cost INS error accumulation in distance estimation using self-resetting. IEEE Trans. Instrum. Meas..

[12] Muhammad M.N., Salcic Z., I. Wang K. Subtractive clustering as ZUPT detector. Proceedings of the IEEE 11th International Conference on Ubiquitous Intelligence and Computing.

[13] Lan K.C., Shih W.Y. (2013). On calibrating the sensor errors of a PDR-based indoor localization system. Sensors.

[14] Antonio R.J., Fernando S., Francisco Z., Jose C.P., Jorge G. (2011). PDR with foot-mounted IMU and ramp detection. Sensors.

[15] Bao H., Wong W.-C. (2014). A Novel Map-Based Dead-Reckoning Algorithm for Indoor Localization. J. Sens. Actuator Netw..

[16] Zengshan T., Yuan Z., Mu Z., Yu L. (2014). Pedestrian dead reckoning for MARG navigation using a smartphone. EURASIP J. Adv. Sign. Process..

[17] Mikov A., Moschevikin A., Fedorov A., Sikora A. A localization system using inertial measurement units from wireless commercial hand-held devices. Proceedings of the International Conference on Indoor Positioning and Indoor Navigation.

[18] Qian J., Ma J., Ying R., Liu P., Pei L. An improved indoor localization method using smartphone inertial sensors. Proceedings of the International Conference on Indoor Positioning and Indoor Navigation.

[19] Shih W.Y., Chen L.Y., Lan K.C. Estimating Walking Distance with a Smart Phone. Proceedings of the Fifth International Symposium on Parallel Architectures, Algorithms and Programming.

[20] Tian Q., Zoran S., I-Kai Wang K., Pan Y. An enhanced pedestrian dead reckoning approach for pedestrian tracking using smartphones. Proceedings of the IEEE Tenth International Conference on Intelligent Sensors, Sensor Networks and Information Processing.

[21] Kang W., Han Y. (2015). SmartPDR: Smartphone-based pedestrian dead reckoning for indoor localization. IEEE Sens. J..

[22] Jin Y., Soh W.S., Motani M., Wong W.C. (2013). A Robust Indoor Pedestrian Tracking System with Sparse Infrastructure Support. IEEE Trans. Mob. Comput..

[23] Lee S., Kim B., Kim H., Ha R., Cha H. (2011). Inertial Sensor-Based Indoor Pedestrian Localization with Minimum 802.15.4a Configuration. IEEE Trans. Ind. Inf..

[24] Ebner F., Deinzer F., Koping L., Grzegorzek M. Robust self-localization using Wi-Fi, step/turn-detection and recursive density estimation. Proceedings of the International Conference on Indoor Positioning and Indoor Navigation.

[25] Zhang R., Xia W., Jia Z., Shen L. The indoor localization method based on the integration of RSSI and inertial sensor. Proceedings of the IEEE 3rd Global Conference on Consumer Electronics.

[26] Edwan E., Bourimi M., Joram N., Al-Qudsi B., Ellinger F. NFC/INS integrated navigation system: The promising combination for pedestrians’ indoor navigation. Proceedings of the International Symposium on Fundamentals of Electrical Engineering.

[27] Ruiz A.R.J., Granja F.S., Prieto H.J.C., Rosas J.I.G. (2012). Accurate Pedestrian Indoor Navigation by Tightly Coupling Foot-Mounted IMU and RFID Measurements. IEEE Trans. Instrum. Meas..

[28] Chirakkal V.V., Park M., Han D.S. (2015). Exploring Smartphone-Based Indoor Navigation: A QR Code Assistance-Based Approach. IEIE Trans. Smart Process. Comput..

[29] Langer M., Kiesel S., Ascher C., Trommer G.F. Deeply Coupled GPS/INS integration in pedestrian navigation systems in weak signal conditions. Proceedings of the International Conference on Indoor Positioning and Indoor Navigation.

[30] Chen L., Hu H. IMU/GPS based pedestrian localization. Proceedings of the 4th Computer Science and Electronic Engineering Conference.

[31] Pratama A.R., Widyawan, Hidayat R. Smartphone-based pedestrian dead reckoning as an indoor positioning system. Proceedings of the International Conference on System Engineering and Technology.

[32] Zhang R., Bannoura A., Hoflinger F., Reindl L.M., Schindelhauer C. Indoor localization using a smart phone. Proceedings of the IEEE Sensors Applications Symposium (SAS).

[33] Gordon N.J., Salmond D.J., Smith A.F.M. (1993). Novel approach to nonlinear/non-Gaussian Bayesian state estimation. IEE Pro. F Radar Sign. Process..

[34] Chateau J., Rousseau P., Albiston G., Cook B., Papanastasiou S., Peytchev E. Implementation and evaluation of particle filtering for indoor positioning. Proceedings of the IEEE Symposium on Computers and Communication (ISCC).

[35] Carpenter J., Clifford P., Fearnhead P. (1999). Improved particle filter for nonlinear problems. IEE Pro. Radar Sonar Navig..

